# Genetic disruption of the baculum compromises the ability of male mice to copulate

**DOI:** 10.1371/journal.pgen.1011787

**Published:** 2025-07-16

**Authors:** Caleb R. Ghione, Nicholas G. Schultz, Sungdae Park, Douglas B. Menke, Matthew D. Dean

**Affiliations:** 1 Molecular and Computational Biology, University of Southern California, Los Angeles, California, United States of America; 2 Pasadena City College, Pasadena, California, United States of America; 3 Department of Genetics, University of Georgia, Athens, Georgia, United States of America; Columbia University, UNITED STATES OF AMERICA

## Abstract

The baculum, a bone in the penis of many mammal species, shows an astonishing level of morphological divergence between species. Despite hundreds of years of interest, biologists have been unable to directly test its function. The goal of the current study is to uncover molecular details that could allow selective disruption of the baculum while allowing normal sexual differentiation and skeletal development. We compare patterns of androgen receptor binding and single cell gene expression in the developing penis, forelimbs and hindlimbs of mice. We identified chondrocytes in all three tissue types, but those from the developing penis show several unique features, including a population of chondrocytes that express both *Runt-related transcription factor 2* (*Runx2*) and *Androgen receptor* (*Ar*). By combining a *Runx2*-Cre allele with a floxed *Ar* allele in mice, we selectively knocked out androgen signaling in late chondrocytes, resulting in a range of defects in baculum morphology. Males with the most disrupted bacula were unable to copulate, and their bacula appears to be disconnected from the corpus cavernosum muscle. Our study provides insights into the diversity of molecular mechanisms leading to bone and offers the first opportunity to directly test the function of the baculum.

## Author summary

The baculum is a morphologically diverse bone that occurs in the penis of many mammal species. It is an unusual bone for many reasons: it diverges rapidly over evolutionary time, it does not form until after birth, and it requires androgen signaling. Although it has been a topic of interest for hundreds of years, its function is almost entirely unknown. Here we characterize the targets of androgen signaling, and gene expression at single cell resolution, during the development of the baculum. Using the power of mouse genetics, we combine insights from our molecular investigations to disrupt the development of the baculum. Males with the most severely disrupted bacula were unable to copulate. Males with the most severely reduced baculum appear to lack connection to the corpus cavernosum muscle, suggesting the ultimate failure to copulate might be related to muscle control.

## Introduction

Reproductive traits evolve rapidly over time, including genitalia and their internal infrastructure, reproductive tracts and the genes they express, and gamete morphology [1–9]. The ultimate driver of this rapid divergence remains enigmatic but could stem from a combination of male-male competition and male-female conflict over fertilization outcomes [10–26].

The baculum is a bone that occurs in the glans penis of many mammal species and shows an astonishing level of morphological divergence having a genetic basis [14,16,27,28]. Many hypotheses have been proposed to explain this rapid divergence, but most investigations have been correlational in nature – for example by testing if/how baculum morphology correlates with the evolution of mating system [11,12,14,15,17–26,29–32]. These studies yielded inconsistent results across mammal orders, perhaps because the baculum has been independently gained at least 9 times and may not be truly homologous. In mice, an experimental evolution study showed that males subjected to sperm competition evolved relatively wide bacula within 27 generations [16]. Similarly, *post hoc* analysis of semi-wild populations of mice showed that males with relatively wide bacula sired more offspring under competitive conditions [11].

These studies demonstrate that baculum morphology influences reproductive fitness, but direct characterization of the function of the baculum requires experimental manipulation of its development. One strategy would be to genetically knock out a critical developmental pathway, but any such approach would have to manipulate development in a tissue-specific manner to avoid off-target disruption of the rest of the skeleton. Fortunately, not all bones express the same genetic network during their formation, providing leverage for bone-specific manipulation. For example, T-box transcription factor 5 (*Tbx5)* is expressed in forelimb, while T-box transcription factor 4 (*Tbx4),* Paired-like homeodomain 1 (*Pitx1)*, are expressed in hindlimb [33–38], even though both *Tbx4* and *Tbx5* target the same fibroblast growth factor gene (*Fgf10)* [39]. Interestingly, limbs and genitals share many aspects of gene expression, and sometimes these are specifically shared between hindlimbs and genitals [33,40]. Disrupting Homeobox protein *HoxD11-13* [41,42] and *Tbx4* [40] altered development of hindlimbs and bacula in mice. In an extreme case, male mice with knocked out transforming growth factor beta receptor I (*Tgfbr1)* developed an extra set of hindlimbs where their genital buds normally develop [43]. Other studies have shown combinations of gene knockouts can produce regional differences in skeletal development. For instance, deletion of transcription factors ALX homeobox protein 1/4 (*Alx1/4)* cause a reduction of the pubis bone, with little effect on pectoral girdle morphology, unless T-box transcription factor 15 *(Tbx15)* and zinc finger protein GLI3 (*Gli3)* are also deleted [44]. On the other end of the spectrum, many “classic” bone genes such as runt-related transcription factor 2 (*Runx2)*, *Osterix (Sp7)*, homeobox protein MSX-2 (*Msx2)*, and *HoxD13* show similar patterns of expression regardless of tissue origin [38,45–54].

Taken together, these studies suggest that, with a better understanding of the heterogeneity of gene expression across skeletal elements, baculum development could be manipulated while avoiding pleiotropic effects. In the case of baculum development, hormonal signaling might be leveraged to achieve this goal. Although hormones are required for proper maintenance after development [55–61] the baculum is unique in that it *requires* androgen to develop [62–70]. Male rats and weasels fail to develop a baculum when castrated neonatally [63–65,67,71–74]; this effect can be reversed by treatment with exogenous testosterone [64,65]. The baubellum, which is a homologous bone that forms in the clitoris of many species [75], undergoes hypertrophy with the administration of testosterone, even in species where the baubellum is normally absent [63,64,67]. The cells that gives rise to the baculum harbors sex steroid receptors [76], and both osteoblasts (the primary bone-forming cells) and osteoclasts (the primary bone-resorbing cells) contain thousands of *Androgen receptor* (*Ar*) RNA per cell [77,78]. Exposing osteoblasts to androgen results in global shifts in gene expression [79], including increased expression of various growth factors such as *Tgf-β* [80,81]. Specifically knocking out *Ar* in mature osteoblasts results in hundreds of genes becoming differentially regulated [82] and a reduction in bone volume [83,84]. *Ar* knockouts demonstrate higher rates of self-renewal among mesenchymal stem cells (the progenitors to bone), suggesting that androgen signaling plays important roles in early stages of cellular differentiation [85]. In cell culture, expression of *Ar* promotes differentiation towards osteoblasts [86]. Interestingly, osteoblasts secrete osteocalcin which upregulates testosterone production [87], revealing feedback mechanisms between a cell’s regulation and androgen signaling.

Taken together, these studies suggest the baculum might be manipulated through androgen signaling. However, the targets of androgen signaling and potential downstream regulation during baculum development remain unknown. Here, we apply modern genomic and transcriptomic methods to fill this gap in knowledge. A combination of chromatin immunoprecipitation targeting androgen receptor (AR ChIP-seq) and single cell RNA sequencing (scRNA-seq) in developing rodent penis and limbs revealed a potential mechanism to specifically disrupt the baculum with minimal side effects. We knocked out *Ar* in a cell-specific manner, by combining a floxed *Ar* with a Cre recombinase driven by a *Runx2* promoter. Male mice showed a range of defective bacula, with minimal off-target effects. Males with the most severely disrupted bacula almost never copulated, as evidenced by lack of copulatory plugs and their inability to sire a litter. The only males to successfully reproduce showed relatively mild disruption of the baculum that retained some of the proximal bulb of the baculum, a region where the corpus cavernosum muscle inserts. Our study indicates muscular control of the baculum might be critical for successful copulation in a male mouse, providing the first direct tests of the function of this enigmatic bone. In addition, our study adds to knowledge of the heterogeneity of bone developmental programs.

## Results

To better understand androgen signaling during bone development and its effect on gene expression, we first determined developmental timepoints where specific bones are beginning to develop. In mice, hindlimbs begin ossifying at embryonic day 15 (E15.5), while the baculum begins to ossify at postnatal day 6 (P6) (S1 Fig). We deployed AR ChIP-seq in hindlimbs of E15.5 mice, penises from P6 and P13 mice, and P2 rat penis (for an interspecific comparison). To link patterns of AR binding to gene expression, we also collected scRNA-seq from E15.5 hindlimbs and forelimbs of mice, and P6 penises from mice. Even though these ages occur before puberty, there is a spike of androgen just prior to birth and AR has a half-life on the order of days [88]. Disrupting androgen signaling prior to puberty impacts known sexual dimorphism in the skeleton [56], indicating androgen signaling is functionally important prior to puberty.

### AR binding is enriched near genes involved in bone and cartilage formation, but only in developing mouse and rat penises

We identified a total of 9,681 AR peaks across the two P6 mouse penis replicates. Of 24,001 protein coding genes annotated in the mouse genome, 4,604 were associated with at least one AR peak. Gene Ontology analyses revealed 12 terms related to osteo- or chondrogenesis that were significantly over-represented among these genes (Table 1). 616 of the 9,681 AR ChIP-seq peaks occurred near a gene associated with functions related to osteo- or chondrogenesis.

**Table 1:** Significantly over-represented Gene Ontology (GO) terms associated with bone development, identified with CHIPENRICH. Rank = ranked by FDR. FDR = False Discovery Rate. N.Genes = number of genes in the genome with that GO annotation. N.with.Peaks = number of those genes that have at least one AR peak. Odds.Ratio = Increased likelihood that an AR peak is associated with a GO annotation, compared to random. BP = Biological Process. CC = Cellular Component. MF = Molecular Function. Numbers in parentheses indicate total number of significantly enriched terms identified. “x” indicates enriched term after re-analysis of 5,476 AR peaks (cutoff 1, see text),  re-analysis of 3,757 AR peaks (cutoff 2, see text), P13 mouse penis, or P2 rat penis.

Rank	Description	FDR	N.Genes	N.with.Peaks	Odds.Ratio	cutoff 1	cutoff 2	P13 mouse	P2 rat
**BP (182 significant)**									
22	extracellular matrix organization (GO:0030198)	0.0001	321	120	2.01	x	x	x	x
23	extracellular structure organization (GO:0043062)	0.0001	322	120	2	x	x	x	x
41	ossification (GO:0001503)	0.0014	421	152	1.7	x	x	x	x
52	cartilage development (GO:0051216)	0.0056	216	88	1.91	x	x	x	x
80	chondrocyte differentiation (GO:0002062)	0.0097	120	59	2.22				x
141	positive regulation of osteoblast differentiation (GO:0045669)	0.0349	79	36	2.38	x	x		x
166	bone development (GO:0060348)	0.0456	257	91	1.63	x			
170	collagen metabolic process (GO:0032963)	0.0476	124	37	2.04			x	
175	positive regulation of ossification (GO:0045778)	0.0485	61	34	2.6	x	x		
**MF (14 significant)**									
1	extracellular matrix structural constituent (GO:0005201)	0.0009	149	66	2.44	x		x	x
**CC (18 significant)**									
1	extracellular matrix (GO:0031012)	0	524	200	2.06	x	x	x	x
3	collagen-containing extracellular matrix (GO:0062023)	0	395	150	2.05			x	x

Assigning AR peaks to a gene is somewhat subjective. Some studies have assumed that AR binding sites within 50 kb of a transcription start site modify that gene’s expression, while others have shown that AR binding can influence the expression of genes more than a million base-pairs away [89–93]. Therefore, we repeated the analysis using a variety of criteria. First, we repeated our analyses after excluding AR peaks that were more than 113,635 bp away from their nearest gene (75% quantile), or with a peak score that was less than 181 (25% quantile), where score is -10*log10qvalue, as calculated by macs2 [94]. This analysis returned 5,476 AR ChIP-seq peaks – approximately half as many peaks as in the full dataset. All terms in Table 1 except three (GO:0002062, GO:0032963, GO:0062023) remained significantly enriched (Table 1). We explored an even more stringent cutoff where we only included peaks that, in addition to these cutoffs, were associated with genes that had at least two AR peaks, reducing our analysis to 3,757 AR ChIP-seq peaks. One additional term (GO:0060348) was lost (Table 1). In short, the enrichment of gene ontology terms associated with osteo- and chondrogenesis was robust to cutoffs.

We also collected AR ChIP-seq data from P13 mouse penises, when the baculum is much further along in development than in P6 males. We identified 3,893 AR ChIP-seq peaks. Of the 12 significantly enriched GO terms from P6 mouse penis, 8 were also identified from P13 penis (Table 1). AR binding is still apparent after 7 more days of development and shows that androgen signaling is a sustained process, even prior to puberty.

For an interspecific perspective, we gathered AR ChIP-seq from P2 rat penises. Interestingly, 9 of the 12 enriched terms observed in P6 mouse penis were also enriched among the rat data (Table 1). Our result indicates androgen signaling shares similarity across these two rodent species, whose most recent common ancestor existed 8–14 million years ago [95].

To compare bone development across different bone systems, we repeated AR ChIP-seq on E15.5 mouse hindlimbs. We found zero AR ChIP-seq peaks in the developing E15.5 mouse hindlimbs, across two biological replicates, even though our controls indicated a successful ChIP-seq (see Methods). This was an unexpected result, as disruption of androgen signaling at E12.5 and E15.5 in mice disrupted digit length ratio in mouse hindlimbs in a previous study [56]. Perhaps the signal of androgen receptor binding in digits is lost when analyzing whole limbs.

To summarize, we identified bona fide AR binding sites across P6 and P13, and P2 rat penises near genes that are known to influence bone and cartilage development. In contrast, we observed no AR binding in E15.5 mouse hindlimbs, suggesting that androgen signaling is a unique aspect of baculum formation that could be exploited with mouse genetic tools.

### Single-cell RNA-sequencing (scRNA-seq) revealed unique populations of chondrocytes in developing mouse penis

**Fig 1:**
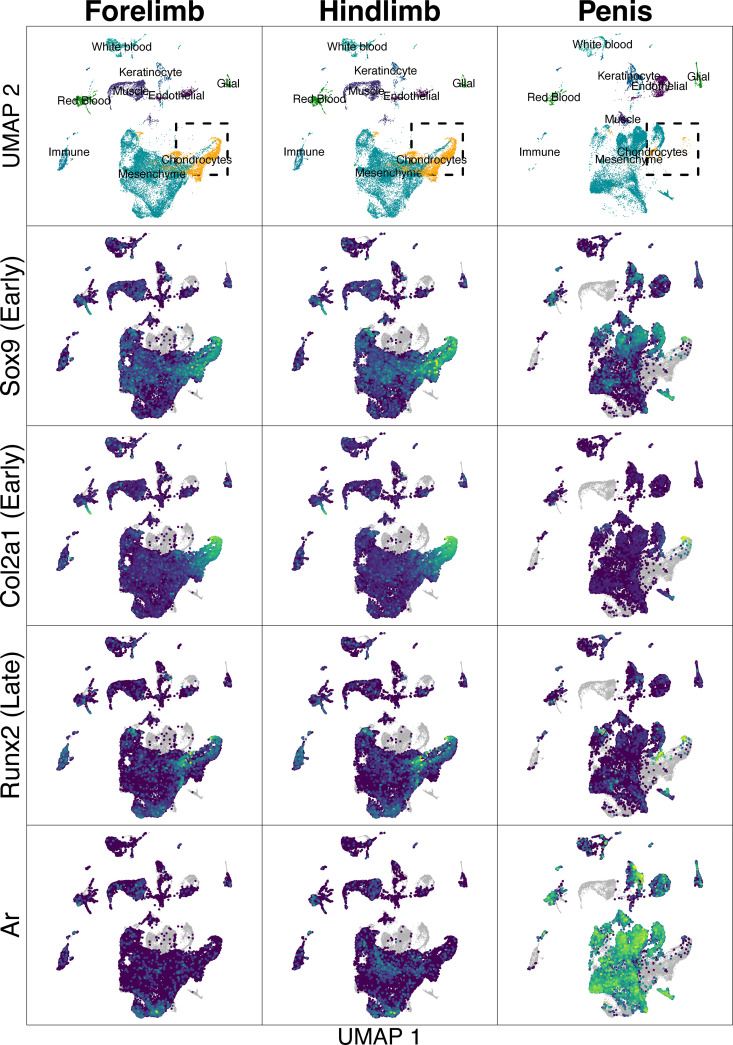
Integrated Data of scRNA-seq data from three tissue types. Top row: general classication of cell clusters across all three samples, determined by inspection of marker genes. Rows 2-5: Expression of *Transcription factor SOX-9* (*Sox9*), *collagen type II alpha 1* (*Col2a1*), *Runx2*, and *Ar*. The words “early” and “late” indicate genes that are turned on early/late in chondrogenesis. Expression levels go from blue (relatively low expression) to yellow (relative high expression). Gray points are "ghost points", indicating cells that were present in one of the other tissues sampled and only included to provide spatial context. Dashed rectangle surrounds all chondrocytes sampled from Penis, and is the area highlighted in Fig 2.

After integrating scRNA-seq data across hindlimb, forelimb, and penis datasets, we identified 40 cell clusters. By analyzing marker genes, we collapsed these into 9 cell types (Fig 1 and S1 Table). Most cells from all three datasets were identified generically as mesenchyme, which makes sense given that much of the tissue we collected probably has not fully differentiated.

**Fig 2:**
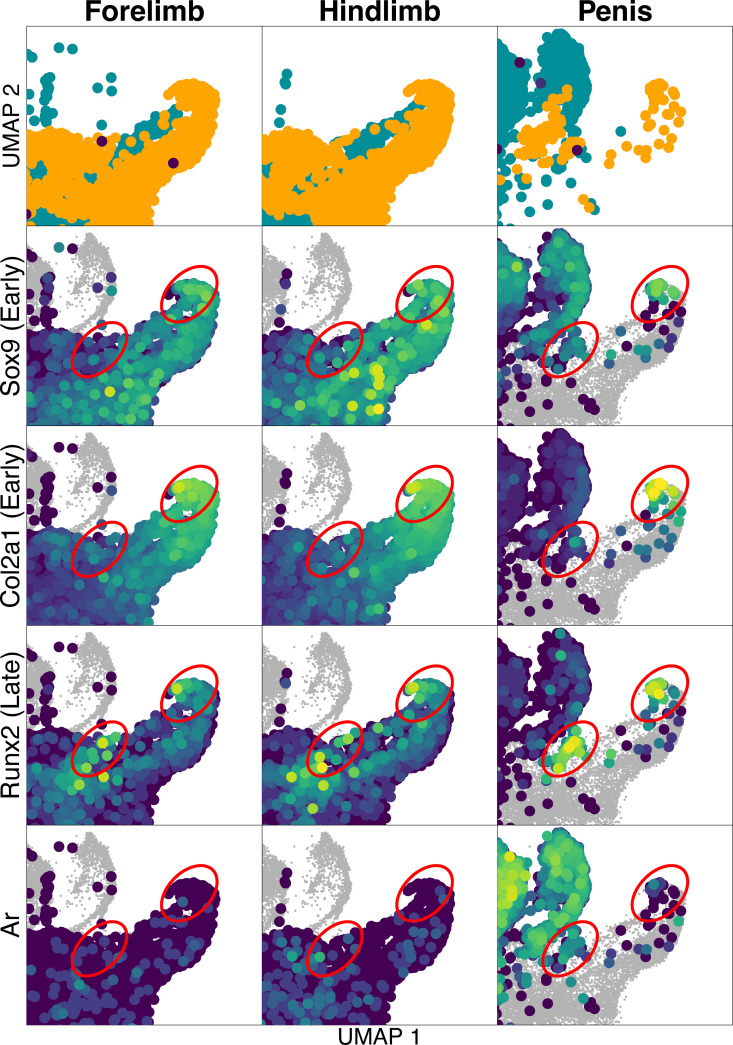
Close-up view of dashed rectangle in Fig 1, including only the area that surrounds all chondrocytes detected from Penis. Rows the same as in Fig 1. The two red circles indicate the two separate clusters of chondrocytes in the penis sample, and corresponding areas in the forelimb and hindlimb. In the penis sample, the upper right circle indicates chondrocyte cells that overlap with forelimb and hindlimb chondrocytes. These chondrocytes do not express Ar. The lower left circle indicates chondrocytes that cluster separately from forelimb and hindlimb chondrocytes and do express Ar.

We identified cells that are part of the osteochondro-lineage across all three tissue types, but those from the developing penis were different than those from limb chondrocytes (Fig 2), albeit UMAP space is difficult to interpret [96]. One group of penis chondrocytes expressed *Sox9*, *Col2a1*, and *Runx2*, but not *Ar* – similar to cells in similar UMAP space in forelimb and hindlimb samples (upper right circles in Fig 2). A second group of chondrocytes expressed *Sox9*, *Runx2*, and *Ar*, but not *Col2a1*, which differed from cells of forelimb and hindlimb samples (lower left circles in Fig 2). *Col2a1* is expressed relatively early in chondrocyte differentiation, while *Runx2* is expressed in later stage chondrocytes as they become hypertrophic (in addition to being expressed in early osteoblasts) [45,97–103]. One interpretation is that these unique penis chondrocytes indicate later stages of chondrogenesis that the forelimb and hindlimb samples have not yet reached. Importantly, it is clear that Runx2 + Ar expression in chondrocytes is unique to the penis.

Interestingly, *Ar* was the most upregulated gene in penis chondrocytes vs. limb chondrocytes (log2 fold-change in penis vs. limb chondrocytes = 6.06, adjusted p ~ 0; Ar detected in 68.5% of penis chondrocytes vs. 2% of limb chondrocytes). Furthermore, 48 of 112 up-regulated genes (log2 fold-change in penis chondrocytes>1, adjusted p < 0.01, proportion of penis cells expressing>50%, proportion of limb cells expressing<50%) showed at least one AR peak in our AR ChIP-seq data collected from developing penis, significantly more than expected from the genome, where 4,604 of 25,190 genes had an AR peak (Χ^2^ = 43.3, df = 1, p-value < 10e-10). Some genes that were both differentially regulated between the penis and limb chondrocytes and had at least one AR peak nearby are involved in matrix development, remodeling processes, and tissue differentiation during bone development, such as *Aspn* and *Lox* [104–106]. In sum, penis chondrocytes uniquely expressed *Ar* in addition to *Runx2* and *Col2a1*.

### Knocking out *Ar* with *Runx2*-Cre disrupts baculum development with minimal side effects

Our results suggest that AR signaling is important in late stages of chondrocyte differentiation in developing penis. Given that AR regulates thousands of genes [107], knocking it out in late chondrocytes could have a large impact on baculum development. We hypothesized that *Runx2* expression could be harnessed to knock out androgen signaling in late chondrocytes of the developing penis. Therefore, we generated male mice that were hemizygous for a floxed *Ar* allele (which is X-linked) and heterozygous for a *Runx2*-Cre allele (homozygous *Runx2*-Cre embryos are inviable).

**Fig 3:**
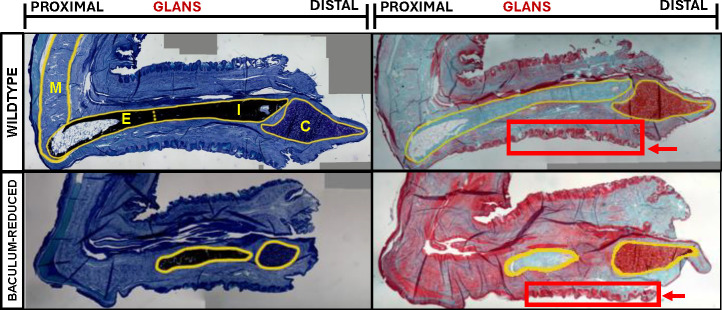
Sagittal sections of a wild type (top row) and baculum-reduced (bottom row) male. Bone is stained black with Von Kossa (left column), while cartilage is stained red with Saf-O (right column). Penises are oriented with distal end to the right. The proximal end of the main bone forms via endochondral ossification (E), while the distal shaft forms via intramembranous ossification (I). The bony baculum is capped by a cartilaginous mass (C) sometimes referred to as the MUMP. The corpus cavernosum muscle (M) engulfs and attaches to the proximal end of the bony baculum. Red boxes and arrows highlight penile spines that surround the penis.

As predicted, these males had under-developed bacula (Fig 3). For simplicity we refer to this male genotype as “baculum-reduced” for the remainder of the manuscript. Their baculum appears as if the proximal end of the baculum, which develops via endochondral ossification [62,64,65,108], is most affected. In baculum-reduced males, the corpus cavernosum muscle, which normally engulfs the proximal end of the baculum [109], appears less developed and detached from the baculum (Fig 3). Towards the distal end of the baculum, there is a small section of bone that appears to be remnants of the distal shaft, which forms via intramembranous ossification [62,64,65,108]. The cartilaginous cap – sometimes called the MUMP [110] – is smaller than normal in baculum-reduced males..

**Fig 4:**
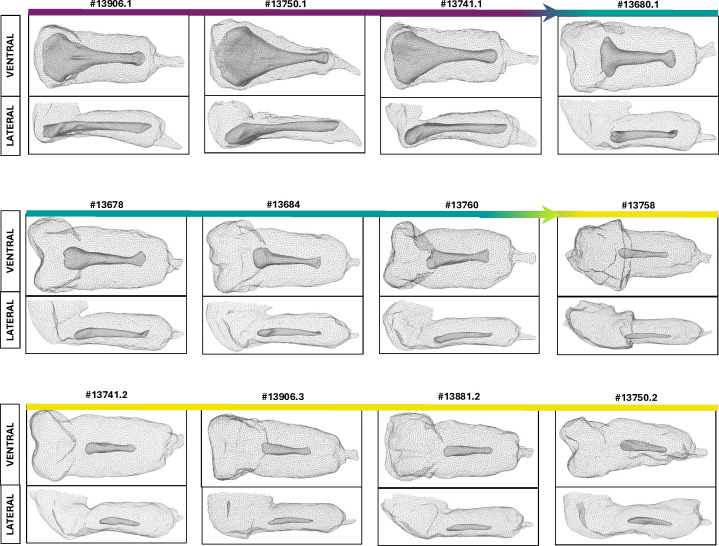
Multi-paneled figure. The panels show microCT scanned images of male animals in our study. The scale ranging from a dark purple to a bright yellow indicates severity of the Androgen Receptor Knock-out (ARKO) phenotype. Dark purple indicates wildtype individuals, turquoise indicates relatively mild ARKO phenotype, and bright yellow indicates the severe ARKO phenotype. Two microCT scans are represented for each individual. The top scan is a dorsal view. The bottom scan is a lateral view. Numbers indiciate specimen ID's.

We produced microCT scans of a larger sample of individuals. Wild type bacula (N = 3, Fig 4) have a wide proximal end that tapers into a distal shaft. Baculum-reduced males range from relatively mild disruption (N = 4 males, Fig 4) to relatively severe disruption (N = 5 males, Fig 4). The most severely disrupted bacula lacked the proximal, bulbous end of the baculum. Combined, their bacula were less than half the length of the three wildtype bacula (Fig 3) (1.52 + /- 0.37 mm versus 3.69 + /-0.20 mm, Wilcoxon Rank Sum Test p = 0.009). Thus, *Runx2*-driven knockout of AR may have selectively disrupted formation of the endochondral region of the baculum, but the disruption was not fully penetrant. It is possible that some cells escape Cre-induced recombination and continue to respond to androgen.

**Fig 5:**
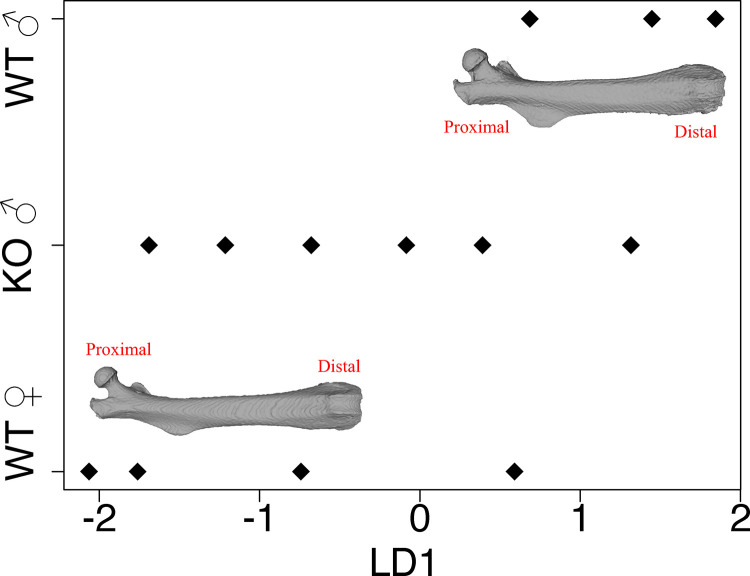
Linear discriminant analysis showing the femora of baculum-reduced males ranges from female-like to male-like. LD1 = linear discriminant dimension 1, calculated by analyzing only wildtype (WT) males and females, and then projecting ARKO (KO) males into that space. Distal ends of femora towards right of figure, proximal towards left.

Apart from the underdeveloped baculum, the rest of the penis seemed relatively normal, including the development of penile spines which are known to require androgen [111] (red boxes and arrows, Fig 3). Although their penises were approximately 85% as long as wildtype penises (3.46 + /- 0.23 mm versus 4.06 + /- 0.25 mm, Wilcoxon Rank Sum Test p = 0.009), this difference could be driven mostly by the absence of approximately half the baculum. Supporting this interpretation, penis length (not including the MUMP) was positively corelated with baculum length across these 12 males (Pearson’s r = 0.8, p = 0.002). In other words, shorter penises might simply result because less space is occupied by a baculum in a reduced male. Other reproductive anatomy including seminal vesical morphology, appeared normal in baculum-reduced males. Interestingly, the femora of baculum-reduced males ranged from “male-like” to “female-like” (Fig 5), indicating that there is at least some feminization of parts of the skeleton, at least in bones that form via endochondral ossification like the femur.

### Reproduction of males with disrupted bacula

All males regardless of genotype behaved normally when paired with females. Immediately upon pairing, males investigated female’s anogenital region and regularly attempted to mount females. We crossed five males to a total of 12 different females for a total of 607 days (Table 2). Only three litters were born (Table 2). In a study with similar crossing data, 740 wildtype male C57BL/6N – a nearly identical genotype to the background studied here (C57BL/6J) – produced 575 litters in 10,233 days of crossing [113]. The number of successful litters produced per cross attempt was significantly lower among baculum-reduced males (Χ^2^ = 15.6, df = 1, p-value < 7.8e-05), and the only two males that successfully sired a litter had relatively mild disruption to their baculum (Table 2). Summarizing Table 2, wildtype males produce 0.056 litters per day of crossing, while baculum-reduced males produce 0.0049 litters per day of crossing. The fact that some baculum-reduced males could sire litters argues against any inherent inability to ejaculate sperm with normal fertilization capacity.

**Table 2:** Crossing results. Wild type data taken from [112]. For all other rows, each row represents a unique male + female cross. Male ID corresponds to Fig 4, unless they were used in histological studies. #days crossed = number of days that males and females were paired. #days plugs checked = number of days that plugs were checked twice daily. Litters born = number of litters born. As an example, male #13678 was paired with two females for 84 days and produced 0 litters. For 31 of these days, the females he was paired with were checked twice a day for copulatory plugs (no copulatory plugs were observed).

Male ID	phenotype	#days crossed	#days plugs checked	Litters born
NA (N = 740)	wildtype	10,233	0	575
#13678	mild	84	31	0
#13678	mild	84	31	0
#13684	mild	84	31	0
#13684	mild	84	31	0
#13680.1	mild	98	0	1
#13680.1	mild	98	0	1
#13760	mild	21	0	1
#13760	mild	21	0	0
#13662	severe	7	7	0
#13662	severe	7	0	0
#13662	severe	5	0	0
#13662	severe	14	7	0

For six of these crosses, we checked for copulatory plugs twice a day, for a total of 607 cross-days. Copulatory plugs are derived from male seminal fluid that coagulates in the vaginal canal and remains visible for 24–48 hours after ejaculation [112–117]. We never observed a copulatory plug, indicating baculum-males often fail to copulate, at least until ejaculation. This could arise from failure to copulate *per se*, or reduced receptivity from females mating to males with disrupted bacula.

## Discussion

Understanding the diversity of ways in which bones develop in nature promises to shed light on the evolution of morphological diversity and potentially guide bioengineering principles. Much of what we know about bone and cartilage development focuses on limb systems [38,118–121] – it is important to expand studies to other skeletal elements.

The baculum evolved at least nine independent times during mammalian evolution [71]. Therefore, the baculum offers an additional opportunity to ask whether independent gains occur through activation of conserved genetic networks, or if independent derivations occur via recruitment of novel pathways. Our study uncovers evidence for both models. On one hand, many “classic” chondrocyte genes – for example *Sox6, Sox9*, and *Col2a1* [45,122,123] are all upregulated early in chondrocytes of developing penis, and all were identified as chondrocyte markers in our data (S1 Table). Inasmuch as the chondrocytes we observe in developing penis give rise to the baculum, then baculum development occurs via activation of conserved genetic networks. On the other hand, some penis chondrocytes formed a distinct cluster with overall divergent gene expression compared to limb chondrocytes, and some of these classic chondrocyte genes were differentially expressed between the two clusters of chondrocytes. The biggest difference was that penis chondrocytes expressed *Ar* at high levels. Future surveys of additional species, covering additional independent derivations of the baculum, will permit more rigorous quantification of whether evolution proceeds by recruitment of existing or novel pathways.

Previous studies documented the requirement of androgen signaling for baculum development [63–65,67]; our study uncovers the first molecular links between those observations and the underlying genetic regulation. Based on the unique expression profile of penis chondrocytes vs. limb chondrocytes, we knocked out androgen signaling to specifically disrupt formation of the baculum. By undertaking the first direct test of the baculum’s function, we showed that males with the most disrupted baculum cannot copulate.

Interestingly, the corpus cavernosum, a major muscle in the penis, attaches to the region of the baculum that is missing from males with the most severe disruptions. The corpus cavernosum is still present in the severe phenotype. However, it does not appear to attach to the proximal end of the baculum (Fig 3). Artificial erections via 10% formalin in bats suggest that the baculum and the corpus cavernosum work in concert to provide support for the penile shaft and distal tip [31]. Males with the most disrupted bacula are entirely missing the proximal region. Although the corpus cavernosum is present, there is no sign that it attaches to the reduced baculum (Fig 3). Therefore, we infer that males use this muscle in conjunction with the baculum during copulation and this process is disrupted in the most severely baculum-reduced males. It should be noted that the corpus cavernosum is also present in species without a baculum, including humans. Future studies involving rabbits, which do not have a baculum and are used heavily as human penile comparison studies [124], could illuminate more of the musculature’s connection with the baculum.

Interestingly, in mice this proximal section of the baculum forms via endochondral ossification, while the distal section forms via intramembranous ossification [62,64,65,108]. Even the most severely reduced bacula seem to retain the distal intramembranous shaft. Therefore, it appears that our Cre-lox approach disrupted the chondrogenesis that occurs during formation of the endochondral proximal section. Additional experiments are required to fully assess this hypothesis, including the use *Col2a1*-Cre (specific to chondrocytes) or *Col10a1*-Cre (specific to hypertrophic chondrocytes) strains of mice.

We have applied modern genomic methods to illuminate the molecular features of baculum development. Our study offers the first direct test of the function of this unusual bone. Males with the most disrupted bacula cannot successfully copulate. This result was unexpected – given the high morphological divergence of the baculum, we predicted that the baculum would function more in some aspect of male-female interactions, for example by contributing to the threshold stimulation required for early embryos to implant. If the baculum functioned solely in the context of erectile support, for example, we would not predict morphological divergence on this scale – instead the baculum should evolve towards some “optimal” structure. Instead, the morphological divergence observed suggests this bone evolves in a non-equilibrium state where optimal shape is continuously shifting, perhaps driven by sexual conflict over fertilization outcomes but not necessarily the ability of a male to copulate. Interestingly, two independent studies mapped paternity success under competitive conditions to the proximal region of the baculum, exactly the same region that seems important in the current study [11,16].

Our main objective was to disrupt the baculum with minimal off-target effects. Combining AR ChIP-seq with scRNA-seq provided a mechanism for doing so. A future goal is to include baculum-reduced males in these molecular investigations in order to identify cell populations that are “missing” and therefore home in more specifically on the cellular basis of baculum development. In addition, expanding molecular studies to other species which have independently gained or lost their bacula could further illuminate the developmental mechanisms of this highly diverse bone.

## Materials and methods

### Ethics statement

All protocols and personnel for mice were approved under the University of Southern California’s Institute for Animal Care and Use Committee, protocols #21218, #21620, and #20584. All mice were of the C57BL/6J genotype and rats were Crl:CD(SD).

### Penis samples

Histology across a developmental time series indicated that in mice the baculum is just beginning to form six days after birth (P6) (S1 Fig). We euthanized three P6 male mice from two different litters (total n = 3) and dissected their penises. We determined sex by observing gonads during dissection. We placed dissected penises in ice cold 1x DPBS for ChIP-seq or DMEM w/o Phenol Red (Hyclone #SH30284.02) for single-cell RNA sequencing. To eliminate some of the extraneous tissue and increase the chance of sampling chondrocytes and osteoblasts, we removed prepuce from each penis, focusing on the glans penis for downstream experiments.

For AR ChIP-seq data only, we dissected developing penises from two additional sources: P13 male mice (n = 6) and P2 rats (n = 2). The P13 sample provided a comparison between developmental timepoints within a species; the P2 rats provided a comparison between species, at a developmental stage that appears equivalent to mouse P6 [64]. Penis tissue was dissected in the same way as for the P6 mouse penis tissue.

### Hindlimb and forelimb samples

Following the well-established Theiler stages, we dissected forelimbs and hindlimbs 15 days after conception, just as ossification is beginning [125]. We paired one male and one female mouse for two weeks, then separated them. We euthanized pregnant females 15 days after copulation, as judged from observation of a copulatory plug. We dissected forelimbs and hindlimbs from male embryos (n = 6), determined by observation of testes.

### Androgen receptor chromatin immunoprecipitation sequencing (AR ChIP-seq)

We incubated penis, forelimb, and hindlimb tissues in Trypsin-EDTA for 5 minutes at 37°C to loosen up the tissue. We inactivated Trypsin-EDTA using DMEM + 10% FBS, then cross-linked DNA to bound proteins by adding 1% formaldehyde, incubated on a horizontal nutator for 20 minutes at room temperature. We quenched the crosslinking reaction using 2.5 M Glycine, spun down tissues at 4,000 rpm for 5 minutes at 4°C. We washed tissues twice in ice-cold PBS, then removed the tissue pellet. We sheared chromatin to a size of 100–300 bp in a bath-based sonicator (Diagenode Bioruptor 300).

Of three different antibodies tested, one (AR rabbit polyclonal, Millipore catalog #06–680; Lot #2967860) produced robust results. We pre-blocked this antibody in PBS/0.5% BSA before incubating overnight with 100 µg of sheared chromatin. We added the reaction to a Protein G Agarose Column (Active Motif, catalog #53037) and incubated for 3 hours. After washing, we eluted chromatin bound to AR antibody from the column and reversed protein-DNA crosslinks by incubating at 65°C overnight. After treating solution with RNase and proteinase K, we purified deproteinated DNA with MicroChIP DiaPure columns (Diagenode, catalog #C03040001).

We produced ChIP and input chromatin control libraries using the NEBNext Ultra II Library Prep Kit (NEB, catalog #E7645S). We performed qPCR to assess enrichment of a positive control site over a negative control site in AR ChIP-seq libraries compared to the input library. As a positive control, we focused on intron5 of *Fkbp5*, which was bound by AR in multiple tissues in previous studies [126,127]. As a negative control, we focused on an intronic region of *Stra8* that lacked matches to known AR binding motifs. The primers used in qPCR were as follows: FKBP5-F: 5’- ACCCCCATTTTAATCGGAGAAC-3’; FKBP5-R: 5’- TTTTGAAGAGCACAGAACACCCT-3’; STRA8control-F: 5’- GGCAGAAGGGTTCATGTGTT-3’; STRA8control-R: 5’- AAATGCTCTCACTTGGCTTGA-3’. We sequenced libraries on the NextSeq 2000 platform at the Georgia Genomics and Bioinformatics Core. Sequencing reads from the ChIP and input control libraries were aligned to the mouse genome (mm10) using BOWTIE 2 v2.4.5 with options “--end-to-end --very-sensitive” [128]. We only kept uniquely aligning reads with fewer than three sequence mismatches to the reference genome.

We observed 88-fold enrichment at the positive control *Fkbp5* site and no enrichment at the negative control site *Stra8* locus [126,127] for P6 mouse penis (S2 Fig). The three top hits from motif analysis revealed predicted sequences of Androgen Response Elements (ARE) and Glucocorticoid Response Elements (GRE) (S2 Fig). The canonical ARE and GRE are not easily distinguished given their sequence similarity. Overall, our results provide confidence that our AR ChIP-seq data identified bona fide AR binding sites.

We identified AR peaks using macs2 v2.7.1 [94] with the options “--bdg --SPMR --qvalue=0.01” and generated fold-enrichment over input control. We performed de novo motif analysis from sequences within 50 bp of AR peak summits, using homer (http://homer.ucsd.edu/), with 100k random genomic sequences as the background. homer uses hypergeometric optimization to detect short sequence motifs that are enriched in sample vs. control [129]. To test enrichment of Gene Ontology annotations with associated AR ChIP-seq peaks, we used the R package chipenrich [130]. In our case, this method assigns AR peaks based on the nearest transcription start site found in the mouse reference genome (mm10), and tests whether the functions associated with those genes are significantly enriched or depleted compared to random expectations using the Benjamini-Hochberg [131] approach to control False Discovery Rate.

From two biological replicates of P6 mouse penises we generated 32 and 41 million total reads, with 26 and 33 million uniquely aligning to the reference genome, respectively. The corresponding control libraries yielded 47 and 54 million total reads, with 37 and 42 million uniquely aligning to the genome, respectively. From two biological replicates of P13 mouse penises, we generated 34 million reads from the control library and 29 million reads from the AR ChIP-seq library. From two biological replicates of P2 rat penises, we generated a total of 45 and 49 million input reads, with 39 and 43 million reads uniquely aligning to the rat reference genome (Rn7) for input versus control libraries. From two input E15 hindlimb libraries we generated 30 and 38 million reads, of which 26 and 33 million uniquely aligned.

### Single cell RNA sequencing (scRNA-seq)

We dissociated tissues using the *gentleMACS Octo Dissociator* using the soft tumor program 37C_m_TDK1. We placed the cell suspension in a MACS SmartStrainer (70 µM) placed in a 15 mL conical tube, then washed it in 10 mL of DMEM. We then centrifuged the sample at 300 G for 7 min. at 4°C, removed the supernatant, and resuspended cells in 100 µL of PBS w/ 0.04% BSA. We processed cell suspensions with a 10X Single Cell 3’ v3.1 kit, aiming to isolate ~10,000 cells, then prepared libraries according to the 10X Genomics protocol, and sequenced using a 200-cycle P3 NextSeq 2000/ run at the Molecular Genomics Core (MGC) at USC Norris Comprehensive Cancer Center, Keck School of Medicine USC aiming for 1.2 billion reads per sample.

We performed alignment, filtering, barcode counting, and Unique Molecular Identifier (UMI) counting using the function cellranger count in the Cell Ranger v7.0.0 software created by 10X Genomics, all aligned to the mm10 version of the mouse genome (NCBI accession number: GCA_000001635.2). After QC and filtering, the P6 mouse penis sequencing data included 19,576 cells averaging 9,606 reads per cell; E15.5 hindlimbs consisted of 20,511 cells averaging 7,251 reads per cell; E15.5 forelimb data consisted of 22,055 cells averaging 6,470 reads per cell.

Based on visual inspection in the R package Seurat 5.1.0, we excluded cells from P6 mouse penis/ E14.5 forelimbs/ E14.5 hindlimbs with fewer than 500/ 100/ 100 reads per cell, more than 6000/ 9000/ 9500 reads per cell, more than 5%/ 10%/ 10% reads derived from mitochondria, or fewer than 0.8/ 0.8/ 0.8 log10 genes detected per UMI. For all three samples, we normalized read counts using the SCTransform function from Seurat, with “method=glmGamPoi” and mitochondrial percentage used as a covariate.

We performed cell clustering and dimensional reduction using the Seurat package in R, in combination with custom scripts. We ran a principal component analysis on the SCTransform‘ed data using the RunPCA function, using the 3000 most variably expressed genes. We clustered cells using the FindClusters function using 50 Principal Components at a resolution of 1. We used the RunUMAP function to project the data into the two-dimensional space of a uniform manifold approximation and projection graph (UMAP) for visualization.

### Comparing chondrocytes

To compare the gene expression profiles of cell types from the three tissues (mouse penis, forelimbs, and hindlimbs) we took a data integration approach via the Seurat package (*v. 5.1.0)* [132]. Integrating data is a strategy to merge multiple datasets to better identify shared cell types. Integration relies on genes that are highly variable across all samples. We integrated the data across tissue type (penis, forelimb, hindlimb) using a custom R script and the IntegrateData function in Seurat (v 5.1.0) [132]. To further compare the gene expression profiles of different cell types in the integrated data sets, we used the FindAllMarkers function in the Seurat package (v 5.1.0) for each of the integrated clusters. We output the top 20 marker genes using the following parameters: an adjusted p-value < 0.01, log2 fold change greater than 1, percentage of cells in the first group expressing a gene greater than 50%, and percentage of cells in the second group expressing a gene less than 10%. Using those marker genes, we were able to identify cell types via primary literature searching.

### *Runx2*-Cre + floxed AR

Our AR ChIP-seq and scRNA-seq data suggested that knocking our AR in chondrocytes or osteoblasts might provide a mechanism to disrupt formation of the baculum. We took a Cre-Lox approach, crossing female mice homozygous for a floxed AR (strain = B6.129S1-Ar^tm2.1Reb^/J, #018450 from The Jackson Laboratory) to male mice expressing Cre-recombinase driven by *Runx2* promoter kindly shared by Dr. Jan Tuckermann at Ulm University in Germany [133]..

To genotype animals with floxed AR, we performed two PCR reactions with two different primers sets. The first primer pair, *Ar* P1 (5′ CAGCACCCTACACTAGAATACTG-3′) and *Ar* P2 (5′-AATGACCTGAGAGTGCTTCCTCC-3′), amplified the 5’ LoxP site. The second primer pair, *Ar* P3 (5′-AGGGCACAGAGTAAGCAGTTTGC-3′) and *Ar* P4 (5′-TCCAGATGTAGGACAGACCTTCC-3′), amplified the 3’ LoxP site. Both primer sets produced a 200- and 220- bp control or floxed allele respectively [134]. We used a touch down PCR to amplify the floxed alleles (94ºC 2 m; 94ºC 20 s; 65ºC 15 s; 68ºC 10 s, reducing the annealing temperature in step 3 by 0.5 ºC every cycle for 28 cycles).

*Runx2*-cre mice had to be maintained as heterozygous because the strain is homozygous lethal. Animals were genotyped with a 3-primer strategy for amplifying the transgene and the wildtype gene: cbfa_24 (5′-CCAGGAAGACTGCCAGAAGG -3′), cbfa_25 (5′-TGGCTTGCAGGTACAGGAG-3′), and cbfa-30 (5′-GGAGCTGCCGAGTCAATAAC-3′). The combination of cbfa_24 and cbfa_25 primers generate a transgene product of 600 bp while the combination of cbfa_24 and cbfa_30 generates a wildtype product of 780 bp. We performed 35 cycles of PCR at 95ºC 1 m; 59ºC 30 s; 72ºC 1 m.

We crossed homozygous floxed AR females with *Runx2*-cre heterozygous males. We expected 50% of male progeny to carry both the hemizygous (*Ar* is X-linked) floxed AR allele and the *Runx2*-cre allele in heterozygous state. The other 50% of males were hemizygous for the floxed allele but homozygous wild type at the *Runx2* allele and were used as controls.

### Histology and microCT scanning

To examine the status of the baculum, we dissected mouse penises at 12 weeks of age, fixed them in formalin for 3–5 days, followed by paraffin embedding, tissue sectioning, and staining with Von Kossa (VK) to quantify mineralization and Safranin-O/Fast Green (SafO/FG) to quantify cartilage. A different set of samples was scanned for microCT scanning. The mouse penis samples for microCT scanning were dissected and fixed in formalin for 3–5 days. To fix tissues, whole mouse penises were put in an Eppendorf tube and submerged in 1 µL of formalin. After fixation, samples were placed in 70% EtOH for 1 day. Samples were placed in rolled up foam and sent to be microCT scanned. Segmentation of the data was done using 3D Slicer (slicer.org) [135] and custom Python and R scripts.

To begin testing for off-target effects on the skeleton, we also microCT-scanned femora from wild type males, females, and baculum-reduced males. Nine landmarks were digitized (S3 Fig), then subjected to Generalized Procrustes Analysis using the gpagen function from the R package geomorph [136]. We subjected the aligned coordinates to linear discriminant analysis using the lda function in the R package MASS [137], using *only* wildtype males and wildtype females. Then, the entire dataset was projected into that space, including femora from baculum-reduced males, using the predict.lda function in R. The intuition was to first define a space that maximally separated wildtype males and females and then project femora from baculum-reduced males to understand whether their femora appear “feminized”.

### Assessing copulatory success

We paired sexually mature males with sexually mature females to monitor for pregnancies. For a subset of crosses, we checked twice daily for copulatory plugs, which is coagulated seminal fluid that remains visible for 24–48 hours following copulation [112–114]. To compare to wild type males, we re-analyzed data taken from C57BL/6N [112], a mouse strain that is nearly genetically identical to the C57BL/6J genetic background of the *Runx2*-Cre and floxed *Ar* strains.

## Supporting information

S1 Fig Histological examination of baculum development in P6 males. Stained with Alizaren Red.
(PDF)

S2 Fig Evidence that we have identified bona fide AR binding sites. A) The top four enriched motifs found among manually curated transcription factor binding sites. The top three hits include known motifs for Androgen Receptor Element (ARE) and Glucocoriticoid Receptor Element (GRE) which is known to share a high degree of sequence similarity. B) Reads mapped from either Control or AR ChIP-seq libraries, with associated AR peak calls. We mapped 88 times as many reads to an intron of *Fkbp5,* an AR-responsive gene known to be bound by AR.
(PDF)

S3 Fig Landmarks taken at maximal/minimal curvatures along femur. Distal at top, proximal at bottom of figure.
(PDF)

S1 Table Top 10 Marker genes used to identify the 10 different cell types. Up-regulated (left half of table) vs. down-regulated (right half of table). Marker genes showed a log2 fold change of at least 1; pc.1 and pct.2 refers to the percentage of focal vs. non-focal cells, respectively, where the gene was detected.
(PDF)
