# Supplementary figures and images for: Genetic disruption of the baculum compromises the ability of male mice to copulate
Source: PLoS Genet. 2025 Jul 16;21(7):e1011787. doi: 10.1371/journal.pgen.1011787 (PMC12313067; doi:10.1371/journal.pgen.1011787)

Supplementary Figure 1

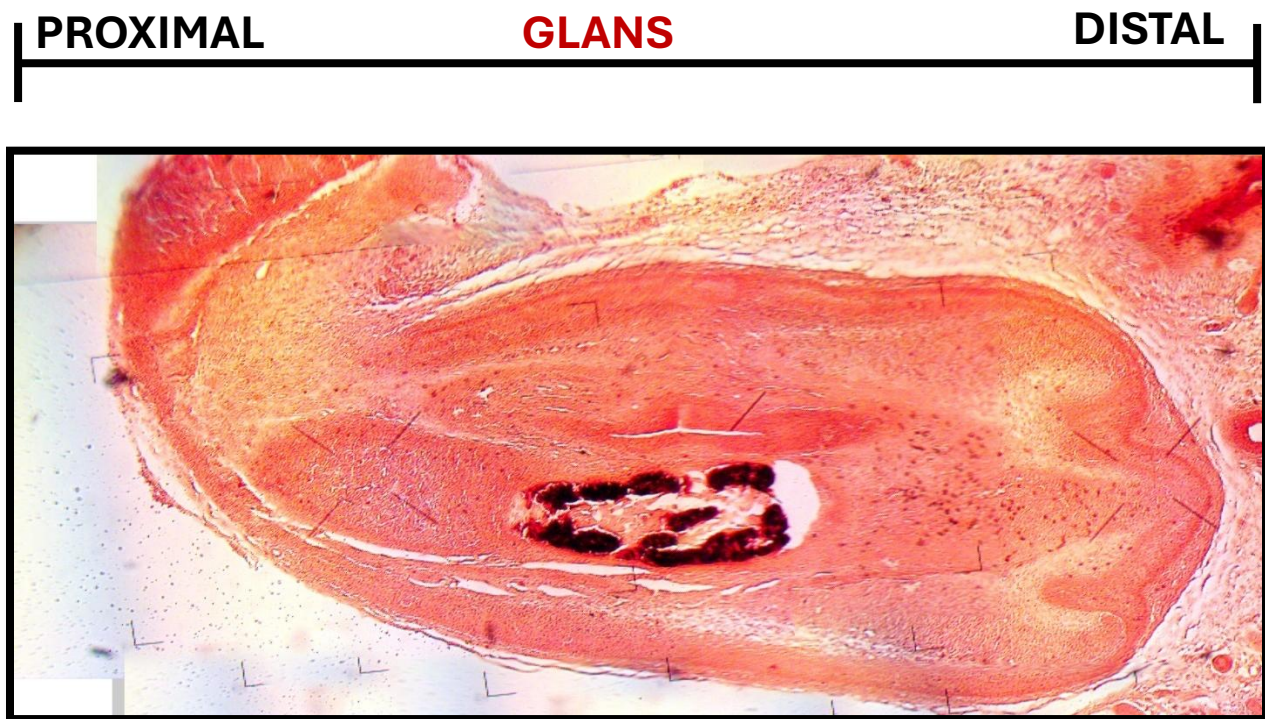

Supplement: S1 Fig — Stained with Alizaren Red. (PDF) [file pgen.1011787.s001.pdf]

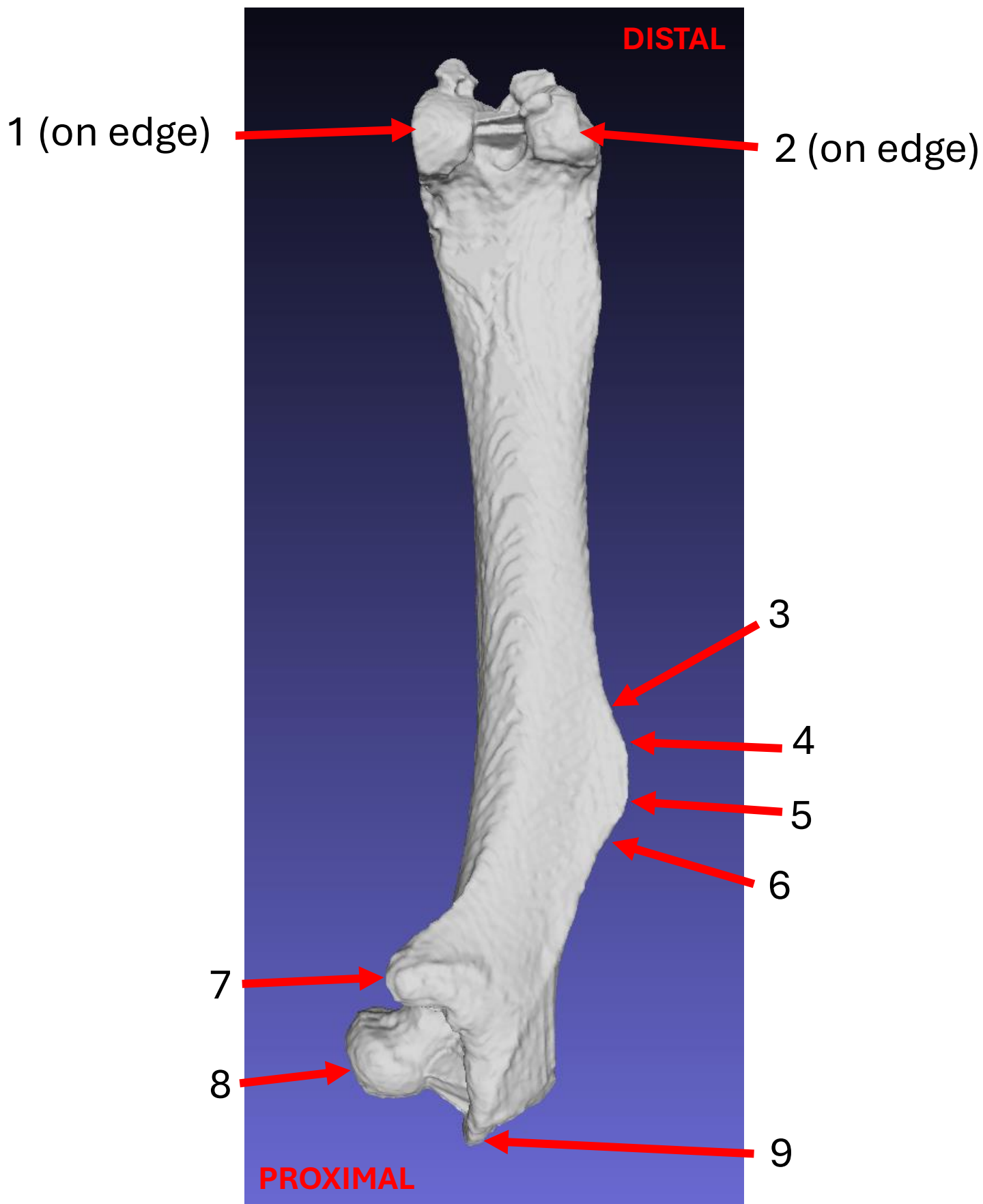

Supplement: S3 Fig — Distal at top, proximal at bottom of figure. (PDF) [file pgen.1011787.s003.pdf]
