# Supplementary material for: Genetic disruption of the baculum compromises the ability of male mice to copulate
Source: PLoS Genet. 2025 Jul 16;21(7):e1011787. doi: 10.1371/journal.pgen.1011787 (PMC12313067; doi:10.1371/journal.pgen.1011787)

Supplementary Figure 2

A

Rank	Motif	Name	P-value
1	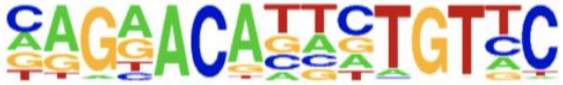	GRE(NR),IR3/RAW264.7-GRE-ChIP-Seq(Unpublished)/Homer	1e-1596
2	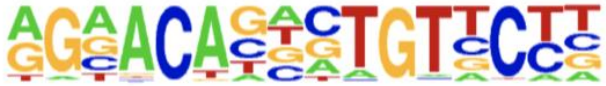	ARE(NR)/LNCAP-AR-ChIP-Seq(GSE27824)/Homer	1e-1593
3	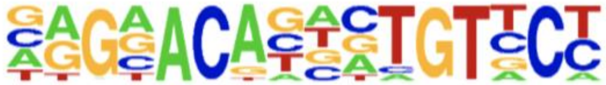	GRE(NR),IR3/A549-GR-ChIP-Seq(GSE32465)/Homer	1e-1561
4	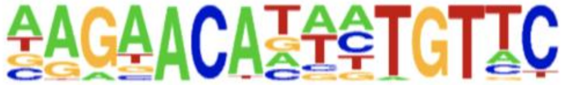	PGR(NR)/EndoStromal-PGR-ChIP-Seq(GSE69539)/Homer	1e-1309

B

Fkbp5 AR peak (chr17)

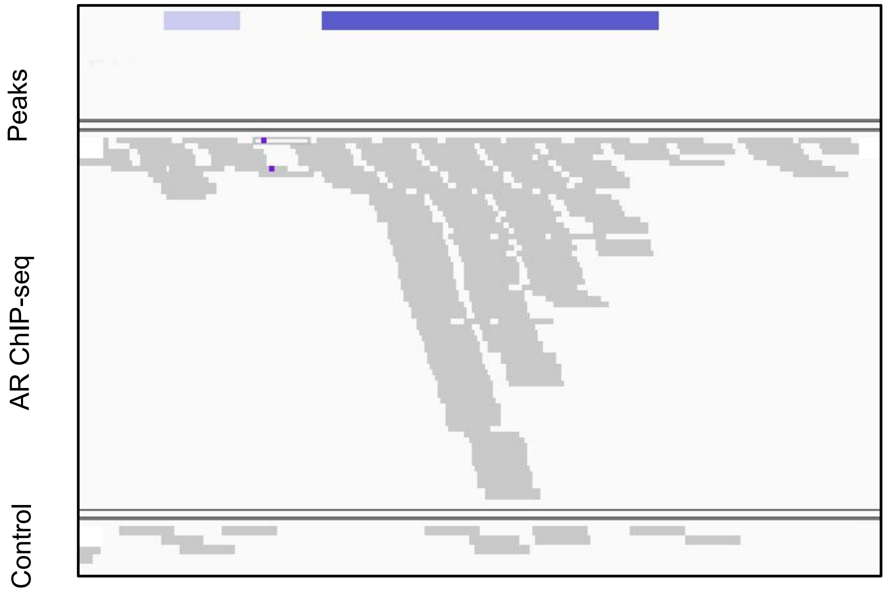

Supplement: S2 Fig — A) The top four enriched motifs found among manually curated transcription factor binding sites. The top three hits include known motifs for Androgen Receptor Element (ARE) and Glucocoriticoid Receptor Element (GRE) which is known to share a high degree of sequence similarity. B) Reads mapped from either Control or AR ChIP-seq libraries, with associated AR peak calls. We mapped 88 times as many reads to an intron of Fkbp5, an AR-responsive gene known to be bound by AR. (PDF) [file pgen.1011787.s002.pdf]
