# Supplementary material for: Genetic disruption of the baculum compromises the ability of male mice to copulate
Source: PLoS Genet. 2025 Jul 16;21(7):e1011787. doi: 10.1371/journal.pgen.1011787 (PMC12313067; doi:10.1371/journal.pgen.1011787)
Supplement: S1 Table — Up-regulated (left half of table) vs. down-regulated (right half of table). Marker genes showed a log2 fold change of at least 1; pc.1 and pct.2 refers to the percentage of focal vs. non-focal cells, respectively, where the gene was detected. (PDF) [file pgen.1011787.s004.pdf]

Supplementary Table 1. Top 10 Marker genes used to identify the 10 different cell types found acr
regulated (left half of table) and down-regulated (right half of table) showed a log2 fold change of r
cells in the focal cell type where that gene was expressed; pct.2 refers to the percentage of cells ir
the log2 fold change in gene expression in the focal vs. non-focal cell types.

gene	avg_log2FC	pct.1	pct.2	p_val_adj	|	gene
					Mesenchyme	
Twist1	2.05	0.7	0.27	0	|	Chd7
Il11ra1	2.02	0.66	0.24	0	|	Sox6
Prrx1	1.62	0.74	0.33	0	|	Msi2
Mfap2	1.57	0.93	0.52	0	|	Cux1
Col6a2	1.54	0.8	0.4	0	|	Elmo1
Fbln1	2.05	0.62	0.23	0	|	Stard13
Cdh11	1.34	0.83	0.44	0	|	Lcp1
Col5a2	1.53	0.91	0.52	0	|	Itga6
Col6a1	1.48	0.85	0.46	0	|	Slc25a5
Pdgfra	1.75	0.56	0.18	0	|	H2-D1
					Keratinocyte	
Krt15	7.79	0.83	0.06	0	|	Vim
Krt5	7.57	0.78	0.04	0	|	Lgals1
Lgals7	6.47	0.86	0.13	0	|	Fbn2
Sfn	6.9	0.75	0.02	0	|	Fstl1
Krt14	7.63	0.74	0.04	0	|	Mfap2
S100a14	7.67	0.7	0.01	0	|	Grb10
Perp	6.85	0.69	0.02	0	|	Tcf4
Fxyd3	6.96	0.63	0.02	0	|	Col1a2
Dsp	6.73	0.62	0.02	0	|	Rrbp1
Krtdap	6.5	0.59	0.04	0	|	Col3a1
					Chondrocytes	
Sox6	3.48	0.72	0.14	0	|	Celf2
Col2a1	5.13	0.73	0.15	0	|	Zeb2
Sox5	3.09	0.76	0.18	0	|	Map1b
Col11a1	3.8	0.85	0.3	0	|	Dpysl3
Mia	4.37	0.55	0.06	0	|	S100a6
Col9a3	4.38	0.59	0.12	0	|	Gsn
Acan	4.66	0.49	0.05	0	|	Nrp1
Col9a2	4.19	0.53	0.09	0	|	Tmem132c
Col9a1	4.25	0.52	0.1	0	|	Ank3
Matn4	4.18	0.49	0.06	0	|	Col4a1

					White blood	
Tyrobp	5.76	0.91	0.04	0	|	Nedd4
Fcer1g	5.32	0.92	0.05	0	|	Rbms3
Lyz2	9.17	0.82	0.02	0	|	Mdk
Laptm5	5.32	0.82	0.03	0	|	Grb10
Lst1	5.57	0.81	0.05	0	|	Auts2
Ptpn18	4.79	0.81	0.06	0	|	Mfap2
Ms4a6c	8.37	0.75	0	0	|	Nfib
Spi1	6.17	0.77	0.02	0	|	Fstl1
Coro1a	5.17	0.78	0.04	0	|	Serpinh1
Csf1r	6.34	0.77	0.03	0	|	Fbn2
					Muscle	
Tnnt1	5.62	0.69	0.06	0	|	Col1a1
Acta2	5.74	0.67	0.06	0	|	Plpp3
Vgll2	6.39	0.57	0.01	0	|	Cdh11
Rbm24	4.96	0.58	0.04	0	|	Mfap2
Tpm2	3.34	0.85	0.33	0	|	Ifitm2
Pdgfa	2.88	0.67	0.15	0	|	Col5a2
Plxna2	2.77	0.64	0.14	0	|	Dcn
Eya4	2.25	0.67	0.16	0	|	Selenom
Tnik	3.31	0.61	0.11	0	|	Igfbp4
Actc1	6.46	0.55	0.06	0	|	Fstl1
					Endothelial	
Cdh5	7.37	0.78	0.01	0	|	Meg3
Emcn	7.79	0.77	0.01	0	|	Mdk
Flt1	5.95	0.79	0.05	0	|	Gpc3
Cd93	7.25	0.73	0.01	0	|	Fbn2
Egfl7	5.28	0.77	0.07	0	|	Rian
Plxnd1	5.28	0.77	0.07	0	|	Ptprd
Esam	6.56	0.71	0.02	0	|	Gas1
Cldn5	6.48	0.69	0.01	0	|	Lrp1
Ecscr	5.6	0.7	0.03	0	|	Col6a1
Ctla2a	5.81	0.7	0.04	0	|	Adgrl3
					Immune	
Arhgap15	5.62	0.96	0.05	0	|	Serpinh1
Srgn	6.22	0.99	0.09	0	|	Nfib
Rac2	5.51	0.92	0.03	0	|	Sparc

Laptm5	4.19	0.88	0.04	0	|	Fstl1
Coro1a	4.23	0.88	0.04	0	|	Mdk
Fcer1g	4.21	0.9	0.06	0	|	Mfap2
Samsn1	6.1	0.85	0.02	0	|	Nfia
Dock2	5.02	0.86	0.03	0	|	Marcks
Tyrobp	3.77	0.88	0.05	0	|	Meg3
Arhgdib	4.05	0.96	0.15	0	|	Tcf4
					Glial	
Plp1	8.28	0.93	0.02	0	|	Ifitm2
Sox10	7.25	0.82	0.01	0	|	Igf2
Erbb3	5.34	0.82	0.03	0	|	Ptprd
Zfp536	4.19	0.91	0.14	0	|	Igfbp4
Fign	4.33	0.87	0.12	0	|	H19
Ednrb	6.5	0.78	0.03	0	|	Gpc3
Gpm6b	3.89	0.92	0.17	0	|	Fbn2
Foxd3	9.2	0.75	0	0	|	Gas1
Cdh19	8.96	0.75	0	0	|	Dlk1
Mpz	11.24	0.74	0.01	0	|	Ebf1
					Red Blood	
Hba-a2	7.48	1	0.42	0	|	Zbtb20
Alas2	6.39	0.56	0.02	0	|	Nfkb1
Hba-a1	7.61	1	0.46	0	|	Iqgap1
Hbb-bt	7.4	0.99	0.47	0	|	Fnbp1
Snca	5.61	0.52	0.02	0	|	Nfe2l2
Mkrn1	3.5	0.58	0.14	0	|	Sat1
Slc25a37	3.99	0.47	0.06	0	|	Ankrd44
Bpgm	3.96	0.45	0.08	0	|	Osbpl8
Ube2l6	4.08	0.31	0.03	0	|	Cgnl1
Tent5c	2.85	0.36	0.09	0	|	Ar

oss our integrated scRNA-seq data (Fig. 1). Up-
nore (less) than 1 (-1). pct.1 refers to the percentage of
ı all non-focal cell types pooled. avg_log2FC refers to

avg_log2FC	pct.1	pct.2	p_val_adj
-3.05	0.05	0.3	0
-2.18	0.12	0.36	0
-1.29	0.36	0.57	0
-1.22	0.28	0.5	0
-2.3	0.15	0.36	0
-2.37	0.13	0.34	0
-5.73	0.01	0.22	0
-2.49	0.09	0.3	0
-1.04	0.44	0.63	0
-3.12	0.07	0.26	0
-2.34	0.57	0.97	0
-2.35	0.58	0.96	0
-2.53	0.43	0.81	0
-2.28	0.5	0.85	0
-2.25	0.46	0.8	0
-1.83	0.5	0.83	0
-2.23	0.57	0.88	0
-2.6	0.58	0.89	0
-1.82	0.61	0.91	0
-2.66	0.61	0.9	0
-2.5	0.3	0.71	0
-1.99	0.28	0.65	0
-1.72	0.29	0.61	0
-1.93	0.22	0.53	0
-1.77	0.34	0.65	0
-2.25	0.18	0.48	0
-2.21	0.14	0.43	0
-3.05	0.08	0.36	0
-1.97	0.16	0.44	0
-1.76	0.31	0.58	0

-2.34	0.42	0.98	0
-2.68	0.29	0.84	0
-2.88	0.33	0.88	0
-2.52	0.3	0.85	0
-2.71	0.39	0.94	0
-2.82	0.27	0.81	0
-2.52	0.35	0.89	0
-2.74	0.33	0.86	0
-2.47	0.39	0.93	0
-2.76	0.3	0.82	0
-2.93	0.42	0.9	0
-2.83	0.18	0.66	0
-2.39	0.26	0.73	0
-2.34	0.36	0.82	0
-1.88	0.48	0.95	0
-1.92	0.36	0.81	0
-3.47	0.23	0.68	0
-2.09	0.23	0.67	0
-2.41	0.35	0.78	0
-1.68	0.44	0.87	0
-2.79	0.43	0.91	0
-2.3	0.41	0.88	0
-2.71	0.32	0.78	0
-2.68	0.37	0.82	0
-2.64	0.23	0.68	0
-2.67	0.35	0.78	0
-2.29	0.32	0.74	0
-2.76	0.11	0.53	0
-2.52	0.31	0.73	0
-3.07	0.25	0.66	0
-3.15	0.23	0.92	0
-3.17	0.22	0.89	0
-3.8	0.26	0.93	0

-3.4	0.2	0.86	0
-3.48	0.22	0.88	0
-3.43	0.16	0.81	0
-3.01	0.23	0.88	0
-2.81	0.25	0.9	0
-2.95	0.27	0.9	0
-2.8	0.26	0.89	0
-2.17	0.54	0.92	0
-3.77	0.16	0.74	0
-2.74	0.25	0.77	0
-2.75	0.26	0.76	0
-3.19	0.24	0.72	0
-2.47	0.31	0.77	0
-2.32	0.4	0.81	0
-2.69	0.25	0.74	0
-3.62	0.15	0.66	0
-3.18	0.18	0.68	0
-1.11	0.57	0.67	0
-1.46	0.19	0.35	0
-1.01	0.25	0.41	0
-1.02	0.16	0.29	0
-1.15	0.17	0.3	0
-1.06	0.19	0.31	0
-1.07	0.17	0.3	0
-1.1	0.26	0.39	0
-1.1	0.14	0.26	0
-1.15	0.16	0.28	0
